# Long‐Term Survival Outcomes of Concurrent Chemoradiotherapy for Postoperative High‐Risk Salivary Gland Carcinomas

**DOI:** 10.1002/cam4.71722

**Published:** 2026-03-25

**Authors:** Shengjin Dou, Xin Wang, Lin Zhang, Wen Jiang, Lulu Ye, Yu Wang, Rongrong Li, Guopei Zhu

**Affiliations:** ^1^ Department of Oral and Maxillofacial‐Head Neck Oncology Shanghai Ninth People's Hospital, Shanghai Jiao Tong University School of Medicine Shanghai China; ^2^ College of Stomatology Shanghai Jiao Tong University Shanghai China; ^3^ Shanghai Center of Head and Neck Oncology Clinical and Translational Science Shanghai China; ^4^ Shanghai Key Laboratory of Stomatology and Shanghai Research Institute of Stomatology Shanghai China; ^5^ National Center for Stomatology and National Clinical Research Center for Oral Diseases Shanghai China; ^6^ Department of Radiation Oncology Shanghai Second People's Hospital Shanghai China; ^7^ Department of Oral Pathology Shanghai Ninth People's Hospital Shanghai Jiao Tong University, School of Medicine Shanghai China

**Keywords:** chemotherapy, high‐risk, postoperative, radiotherapy, salivary gland carcinoma

## Abstract

**Background:**

This study evaluated the long‐term survival outcomes of concurrent chemoradiation (CCRT) for high‐risk salivary gland carcinomas (SGCs).

**Method:**

Postoperative patients with high‐risk SGCs, other than adenoid cystic carcinoma (ACC), with T3‐4/N1‐3M0 disease were enrolled. This study included a cohort of 55 patients who received CCRT, derived from a prospective phase II trial, and a retrospective cohort of 61 patients treated with RT alone.

**Results:**

The median follow‐up for survivors was 54.1 months. In the subgroup analysis, patients without ENE treated with CCRT showed significantly better 5‐year OS (83.4% vs. 69.0%, *p* = 0.032), with a numerically higher 5‐year DFS (57.3% vs. 41.6%, *p* = 0.062). In the subgroup of patients with N0‐1 disease, those treated with CCRT showed a numerically higher 5‐year DFS (69.1% vs. 44.9%, *p* = 0.073) and OS (90.7% vs. 76.2%, *p* = 0.057). On multivariate analyses, CCRT significantly predicted superior DFS (*p* = 0.021) and OS (*p* = 0.004) for patients without ENE and superior DFS (p = 0.027) for patients with N0‐1 disease.

**Conclusion:**

For postoperative high‐risk non‐ACC SGCs, CCRT was associated with improved long‐term survival outcomes in patients without ENE or with N0‐1 disease, which need further evaluation in randomized trials. However, for patients with ENE or N2‐3 disease, they may need alternative treatment strategies to enhance their prognosis.

**Trial Registration:**

The prospective cohort analyzed in this study originated from the non‐ACC group enrolled in a phase 2 clinical trial (NCT02776163)

## 1 Introduction

Salivary gland carcinomas (SGCs) comprise malignancies of the major (e.g., sublingual, submandibular, and parotid) and the minor salivary glands. They are rare and account for approximately 3% of all head and neck cancers [1]. SGCs are characterized by a variety of pathological types and exhibits significant heterogeneity [2, 3]. Surgical resection is the major therapeutic approach for SGCs, and postoperative radiotherapy(RT) is administered to patients presenting with adverse pathologic risk factors [4, 5]. However, despite the adoption of adjuvant radiotherapy, locoregional failure rates reported was still exceed 30% and distant failures were also common, particularly in high‐risk cases [6, 7, 8].

Whether postoperative concurrent chemoradiotherapy (CCRT) should be administered to high‐risk patients is still a subject of ongoing debate [9, 10, 11, 12, 13, 14, 15, 16, 17, 18]. Although high‐quality evidence remains limited, CCRT is widely implemented in clinical practice. However, most studies [11, 12, 13, 15, 17] were single‐institution, retrospective analyses with small sample sizes and varying chemotherapy regimens. Two retrospective studies with large cohorts of patients from the National Cancer Database [16, 19] compared survival between adjuvant CCRT and RT alone; postoperative CCRT did not improve overall survival (OS) in propensity‐matched analysis or multivariable Cox regression. A multicenter retrospective analysis of 411 postoperative salivary gland carcinoma (SGC) patients, who received RT alone (*n* = 263) or CCRT (*n* = 148), demonstrated that POCRT was associated with significantly better long‐term OS and progression‐free survival (PFS) in patients with lymph nodal metastasis [18]. However, these conclusions were limited by the retrospective design, database biases, and the heterogeneity of chemotherapy regimens, emphasizing the need for high‐quality prospective data to better define the role of chemotherapy.

Prospective investigations assessing the impact of CCRT in postoperative high‐risk SGCs remain limited. RTOG 1008 (NCT01220583) is an active, randomized phase II/III trial evaluating the efficacy of CCRT versus RT alone in high‐risk resected SGCs. The trial employs a weekly cisplatin regimen at 40 mg/m^2^ as a single agent, with the primary goal of enhancing PFS by decreasing locoregional recurrence. Given that both distant metastasis and locoregional recurrence are frequent failure patterns in major [7] and minor SGCs [6, 8], the use of concurrent chemotherapy with multiple agents might offer a promising alternative. Aside from cisplatin, taxanes appear to be active in histologies other than adenoid cystic carcinoma (ACC) [10]. Our previous study also suggested that SGCs are sensitive to docetaxel, which may offer a benefit in overcoming radioresistance [20]. Therefore, the aim of this study was to evaluate the efficacy and safety of postoperative CCRT using a combination of docetaxel and nedaplatin (TP) in high‐risk non‐ACC SGCs patients.

## 2 Patients and Methods

### 2.1 Patients

The study was approved by the Local Ethics Committee (2016147) and conducted in accordance with the principles of the Declaration of Helsinki. It included a prospective cohort treated with CCRT and a retrospective cohort treated with RT alone. The prospective CCRT cohort was a non‐ACC cohort of a phase II trial, which is registered with ClinicalTrials.gov (https://clinicaltrials.gov/study/NCT02776163). Postoperative patients with pathologically confirmed non‐ACC SGCs were enrolled in the prospective CCRT cohort, while those receiving retrospective RT alone were collected based on the same inclusion criteria. The eligibility criteria were as follows: ① Participants aged between 18 and 75 years; ② Tumor stage T1‐2 and nodal stage N1‐3, or tumor stage T3‐4 and nodal stage N0‐3, following radical surgical resection (based on the 7th edition of the AJCC staging system, 2017); ③ Histologically confirmed grade III SGC, or grade II SGC with R2 resection or N1‐3 disease; ④ Karnofsky Performance Status (KPS) score of 70 or higher; ⑤ No signs of distant metastasis; ⑥ Expected survival of at least 6 months. Exclusion criteria included patients who had only undergone biopsy, had a history of malignant cancer (except for non‐melanomatous skin cancer) within the past 5 years, or had significant underlying medical conditions. The histologies included in the study were as follows: salivary duct carcinoma (SDC), G2‐3 carcinoma ex‐pleomorphic adenoma (Ca ex PA), G2‐3 mucoepidermoid carcinoma (MEC), grade 3 adenocarcinoma not otherwise specified (NOS), and G2‐3 other histologies. Lymphoepithelial carcinoma, carcinosarcoma, and neuroendocrine carcinoma were excluded. All pathology diagnoses were confirmed through a combination of histomorphology, immunohistochemistry (IHC), fluorescence in situ hybridization (FISH), and, where applicable, other molecular analyses. All histopathological diagnoses are based on the WHO Classification of Tumors. SDC and poorly differentiated carcinoma are classified as grade 3. For MEC, grading follows the AFIP system [21]. The grading of Ca ex PA is determined by both the carcinoma type and the degree of invasion. For adenocarcinoma, NOS, and other variants, the grade is assessed based on several factors, including nuclear pleomorphism, mitotic activity, necrosis, perineural invasion, lymphovascular invasion, and bone involvement. To assess for residual disease, surgeons relied on intraoperative observations, analysis of resection margins, and postoperative imaging—including MRI, CT, and 18F‐FDG PET/CT scans of the head and neck. Patients who met the inclusion criteria and were treated with RT alone were retrospectively included.

### 2.2 Study Treatment

All patients received postoperative RT, which was planned and delivered using intensity‐modulated radiation therapy (IMRT). Treatment simulation involved computed tomography (CT) and immobilization with a custom thermoplastic head–neck–shoulder mask. The clinical target volumes (CTVs) were delineated as follows: CTV1 included the macroscopic residual tumor and/or tumor bed; CTV2 extended to cover CTV1 along with typical pathways of spread and high‐risk nodal levels; and CTV3 included low‐risk nodal regions. Planning target volumes (PTVs) were delineated by uniformly expanding CTV1 by 5 mm and CTV2 and CTV3 by 3 mm. The prescribed doses were 66–70 Gy for PTV1, 60 Gy for PTV2, and 54 Gy for PTV3, administered in 30–32 fractions.

Patients in the CCRT group underwent two cycles of chemotherapy, delivered every 3 weeks commencing on day 1. The regimen consisted of intravenous docetaxel at 70 mg/m^2^ on day 1 and nedaplatin at 35 mg/m^2^ on days 1 and 2. All patients received standard prophylactic antiemetic therapy to address docetaxel‐induced side effects. To prevent hypersensitivity reactions, skin toxicity, and fluid retention, oral dexamethasone 8 mg was prescribed twice daily for three consecutive days prior to chemotherapy initiation. Chemotherapy was only given to patients with adequate bone marrow, hepatic, and renal function. If a delay exceeding 2 weeks occurred, chemotherapy was discontinued. No primary prophylactic granulocyte colony‐stimulating factor or antibiotics were used.

### 2.3 Evaluation

Before enrollment, all patients provided a detailed medical history and underwent physical exams to assess their eligibility. Patients underwent comprehensive baseline assessments, including full blood count (FBC), biochemical profiling, and contrast‐enhanced imaging (MRI or CT) of the head and neck region, supplemented by additional staging investigations as clinically indicated. Surgical resection completeness was assessed using the R0‐1‐2 criteria, and any residual disease was evaluated by a multidisciplinary team. During treatment, patients were monitored at least weekly. Follow‐up visits occurred every 3 months during the first 2 years post‐radiotherapy and every 6 months from the third to fifth years.

### 2.4 Study Design and Analysis

The prospective cohort was sourced from a single‐arm, phase II clinical trial designed to assess the efficacy and safety of integrating TP chemotherapy with IMRT for the management of postoperative high‐risk SGCs. To examine long‐term survival outcomes, a retrospective cohort treated with RT alone was included for comparison. The primary endpoint for the prospective cohort was the 5‐year disease‐free survival (DFS). Based on previous research, the DFS rate for stage III‐IV patients treated with RT alone was estimated to be around 50%. To assess whether CCRT could enhance the 5‐year DFS rate to 75%, a sample size calculation determined that 49 patients were required to achieve 80% statistical power. This would enable detection of an improvement from 50% to 75% using a two‐sided log‐rank test with a significance level (α) of 0.05. Taking into account a 10% follow‐up loss over 2 years, at least 54 evaluable patients were needed for the non‐ACC group. Secondary endpoints included OS, locoregional relapse‐free survival (LRRFS), distant metastasis‐free survival (DMFS), and toxicity.

Toxicity was evaluated according to the National Cancer Institute Common Terminology Criteria for Adverse Events (version 4.03). Time‐to‐event data were calculated from the date of surgery to the last follow‐up or death. Univariate analysis was performed to identify clinical prognostic factors associated with survival. Subsequently, multivariate analysis using a Cox proportional hazards regression model with a stepwise method was conducted to identify independent predictors of survival. In order to maximize control over known confounders, the multivariable models were constructed primarily based on clinical considerations and established literature. To ensure consistency and transparency, we standardized our modeling strategy: for all subgroup analyses, the multivariable Cox regression models include the same set of clinically relevant covariates as used in the main analysis, with the exception that the subgroup‐defining variable is excluded from the model when analyzing the respective subgroup. All tests were two‐tailed (significance level: *α* = 0.05). Statistical analyses were performed using SPSS for Windows (version 23.0; SPSS Institute, IBM, US) and GraphPad Prism (version 9; GraphPad Software, US).

## 3 Results

### 3.1 Patient Characteristics

Between May 2016 and August 2022, a total of 55 patients were enrolled in the prospective cohort, and between March 2018 and October 2023, 61 patients were included in the retrospective cohort. The patient characteristics are presented in Table 1. No significant differences were observed between the baseline characteristics of the two cohorts. The CCRT cohort showed a tendency toward a higher proportion of patients with T1‐2 and N2‐3 stages (*p* = 0.075 and *p* = 0.094, respectively).

**TABLE 1:** Patient clinicopathological characteristics.

Characteristics	CCRT (*N* = 55)	RT alone (*N* = 61)	*p*
Gender			
Male	**42 (76.4%)**	**46 (75.4%)**	**0.905**
Female	**13 (23.6%)**	**15 (24.6%)**	
Age at diagnosis, years			
Range	**18–75**	**30–74**	**0.151**
Median	**56**	**61**	
Subsite			
Major SGCs	**41 (74.5%)**	**50 (82.0%)**	**0.332**
Minor SGCs	**14 (25.5%)**	**11 (18.0%)**	
Grade			
2	**10 (18.2%)**	**11 (18.0%)**	**0.983**
3	**45 (81.8%)**	**50 (82.0%)**	
Pathology			
Ca ex PA	**31 (56.4%)**	**29 (47.5%)**	**0.712**
MEC	**11 (20.0%)**	**13 (21.3%)**	
Adenocarcinoma, NOS	**8 (14.5%)**	**9 (14.8%)**	
SDC	**3 (5.5%)**	**4 (6.6%)**	
Others	**2 (3.6%)**	**6 (9.8%)**	
T Stage			
T1‐2	**12 (21.8%)**	**6 (9.8%)**	**0.075**
T3‐4	**43 (78.2%)**	**55 (90.2%)**	
N Stage			
N0‐1	**23 (41.8%)**	**35 (57.4%)**	**0.094**
N2‐3	**32 (58.2%)**	**26 (42.6%)**	
Stage			
III	**9 (16.4%)**	**11 (18.0%)**	**0.812**
IV	**46 (83.6%)**	**50 (42.6%)**	
LVI			
No	**49 (89.1%)**	**51 (83.6%)**	**0.392**
Yes	**6 (10.9%)**	**10 (16.4%)**	
PNI			
No	**30 (54.5%)**	**30 (49.2%)**	**0.564**
Yes	**25 (45.5%)**	**31 (50.8%)**	
ENE			
No	**44 (80.0%)**	**50 (82.0%)**	**0.787**
Yes	**11 (20.0%)**	**11 (18.0%)**	
Surgery status			
R0	**38 (69.1%)**	**40 (65.6%)**	**0.789**
R1	**12 (21.8%)**	**13 (21.3%)**	
R2	**5 (9.1%)**	**8 (13.1%)**	

Abbreviations: Ca ex PA, Carcinoma ex pleomorphic adenoma; CCRT, concurrent chemoradiotherapy; ENE, extranodal extension; LVI, lymph‐vascular invasion; MEC, Mucoepidermoid carcinoma; NOS, not otherwise specified; PNI, perineural invasion; RT, radiotherapy; SDC, Salivary duct carcinoma; SGC, Salivary gland carcinoma.

### 3.2 Survival Outcomes

**FIGURE 1:**
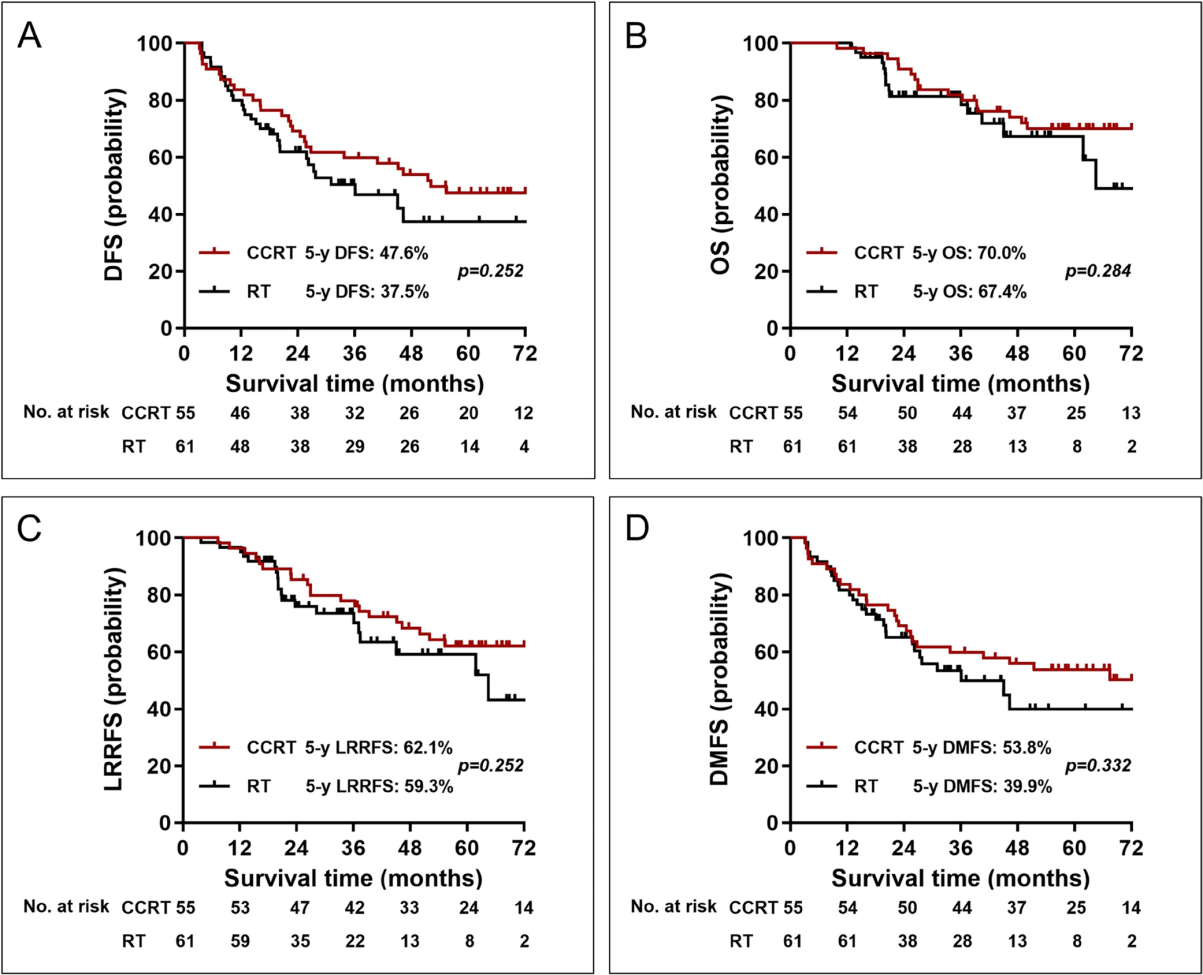
Kaplan–Meier curves between CCRT cohort and RT alone cohort. (A) DFS, (B) OS, (C) LRRFS, and (D) DMFS. Abbreviations: CCRT, concurrent chemoradiotherapy; RT, radiotherapy; DFS, disease‐free survival; OS, overall survival; LRRFS, locoregional recurrence‐free survival; DMFS, distant metastasis‐free survival.

At the end of the study, 85 (70.2%) patients were alive, with a median follow‐up of 54.1 months for survivors. The median follow‐up was 58.7 months and 34.7 months for CCRT cohort and RT alone cohort, respectively. The estimated 5‐year DFS rate was 47.6% for CCRT cohort, and 36.9% for RT alone cohort (*p* = 0.207). The estimated 5‐year OS, LRRFS, DMFS rates of the CCRT cohort were 70.0%, 62.1%, and 53.8%, respectively, which were no difference compared with RT alone cohort (Figure 1).

### 3.3 Univariate and Multivariate Analyses of All Patients

The results of univariate and multivariate analyses for all patients were presented in (eTable S1 and Table 2), respectively. Notably, CCRT did not show an association with improved DFS, OS, LRRFS, or DMFS outcomes in either analysis. On the other hand, univariate analyses revealed that only ENE and N2‐3 disease were predictors of both lower DFS (5‐year DFS: 49.6% vs. 8.6%, *p* = 0.000 and 56.4% vs. 27.9%, *p* = 0.002, respectively) and OS (5‐year OS: 76.5% vs. 30.5%, *p* = 0.000 and p = 0.002, respectively). These findings were further corroborated by multivariate analysis (DFS: *p* = 0.016 and *p* = 0.070; OS: *p* = 0.024 and *p* = 0.013, respectively), identifying a subgroup of patients at very high risk.

**TABLE 2:** Multivariable analysis of all patients (*).

All patients (*n* = 116)		DFS	OS	LRRFS	DMFS
		HR (95% CI), *p*	HR (95% CI), *p*	HR (95% CI), *p*	HR (95% CI), *p*
**CCRT**	**Yes vs No (ref)**	0.647 (0.362–1.155) *p* = 0.141	0.671 (0.313–1.438) *p* = 0.305	0.607 (0.304–1.212) *p* = 0.157	0.688 (0.381–1.243) *p* = 0.216
**Sex**	**Female vs Male (ref)**	1.171 (0.537–2.557) *p* = 0.691	**3.881 (1.182–12.751) *p* = 0.025** *	1.985 (0.693–5.687) *p* = 0.202	1.388 (0.619–3.114) *p* = 0.426
**Age (years)**	**65 vs ≤ 65 (ref)**	0.709 (0.347–1.447) *p = 0.345*	1.035 (0.404–2.652) *p = 0.943*	1.089 (0.483–2.456) *p = 0.837*	0.563 (0.255–1.239) *p = 0.153*
**Site**	**Minor vs Major (ref)**	0.864 (0.350–2.134) *p = 0.751*	**0.077 (0.014–0.437)** ***p =* 0.004** *	**0.343 (0.099–1.188)** ***p = 0.091***	0.556 (0.222–1.391) *p = 0.209*
**T stage**	**T3‐4 vs T1‐2 (ref)**	0.776 (0.329–1.830) *p = 0.563*	0.651 (0.216–1.964) *p = 0.446*	0.661 (0.240–1.821) *p = 0.423*	0.698 (0.279–1.742) *p = 0.440*
**N stage**	**N2‐3 vs N0‐1 (ref)**	**2.200 (1.158–4.178)** ***p* = 0.016** *	**3.478 (1.182–10.232)** ***p* = 0.024** *	**4.160 (1.698–10.195)** ***p = 0.002*** *	1.741 (0.890–3.405) *p = 0.105*
**Grade**	**III vs II (ref)**	**2.021 (0.880–4.640)** ***p* = 0.097**	1.072 (0.384–2.993) *p = 0.894*	1.431 (0.535–3.833) *p = 0.475*	1.843 (0.795–4.274) *p = 0.154*
**Surgery Status**	**R2 vs R0R1 (ref)**	**2.201 (1.026–4.723) *p* = 0.043** *	1.536 (0.458–5.145) *p* = 0.487	1.720 (0.614–4.815) *p* = 0.302	**2.412 (1.105–5.264) *p* = 0.027** *
**ENE**	**Yes vs No (ref)**	**1.949 (0.947–4.011) *p* = 0.070**	**3.075 (1.264–7.480) *p* = 0.013** *	1.773 (0.797–3.943) *p* = 0.161	**2.672 (1.269–5.629) *p* = 0.010** *
**LVI**	**Yes vs No (ref)**	0.751 (0.540–2.349) *p* = 0.751	0.971 (0.390–2.417) *p* = 0.949	1.830 (0.835–4.009) *p* = 0.131	0.951 (0.440–2.060) *p* = 0.899
**PNI**	**Yes vs No (ref)**	1.431 (0.796–2.572) *p* = 0.231	1.138 (0.533–2.431) *p* = 0.738	1.271 (0.641–2.519) *p* = 0.492	1.312 (0.721–2.387) *p* = 0.374

Abbreviations: CCRT, concurrent chemoradiotherapy; DFS, disease‐free survival; DMFS, distant metastasis‐free survival; ENE, extranodal extension; LRRFS, locoregional recurrence‐free survival; LVI, lymph‐vascular invasion; OS, overall survival; PNI, perineural invasion; ref., reference; RT, radiotherapy.

* Multivariable analysis included all the variables in this table.

### 3.4 Subgroup Analysis

**FIGURE 2:**
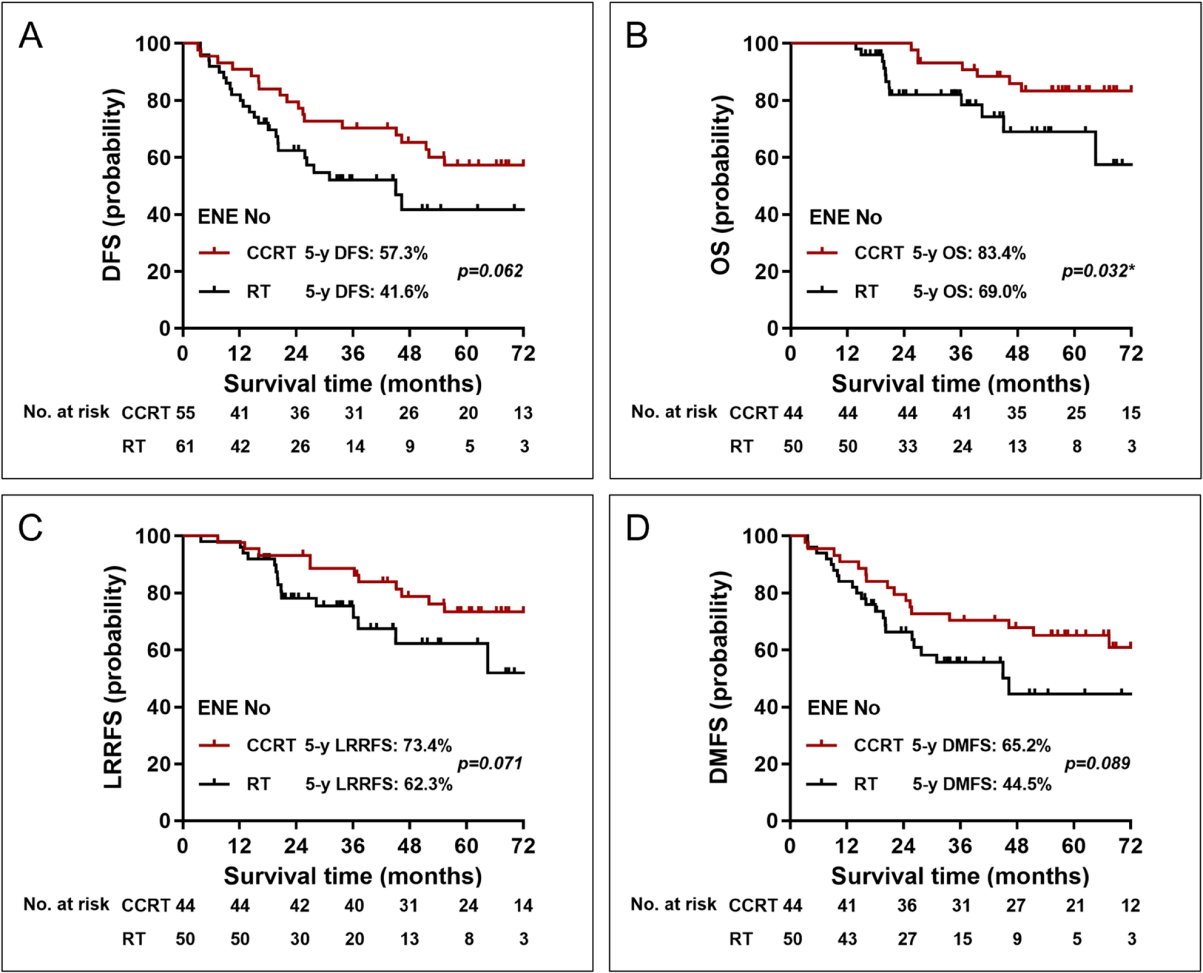
Kaplan–Meier curves between CCRT and RT alone in patients without ENE. (A) DFS, (B) OS, (C) LRRFS, and (D) DMFS. Abbreviations: CCRT, concurrent chemoradiotherapy; RT, radiotherapy; ENE, extranodal extension; DFS, disease‐free survival; OS, overall survival; LRRFS, locoregional recurrence‐free survival; DMFS, distant metastasis‐free survival.

To determine which patient groups might benefit from concurrent chemotherapy, we conducted subgroup analyses within high‐risk populations. In the very high‐risk subgroup with ENE or N2‐3 disease, there was no significant difference in survival outcomes between the CCRT and RT alone cohorts (Table S2). However, CCRT demonstrated a survival benefit in certain high‐risk subgroups. In patients without ENE, those treated with CCRT had a significantly higher 5‐year OS rate (83.4% vs. 69.0%, *p* = 0.032). Additionally, CCRT showed a numerically better 5‐year DFS (57.3% vs. 41.6%, *p* = 0.062), 5‐year LRRFS (73.4% vs. 62.3%, *p* = 0.071), and 5‐year DMFS (65.2% vs. 44.5%, *p* = 0.089) compared to RT alone (Figure 2).

**FIGURE 3:**
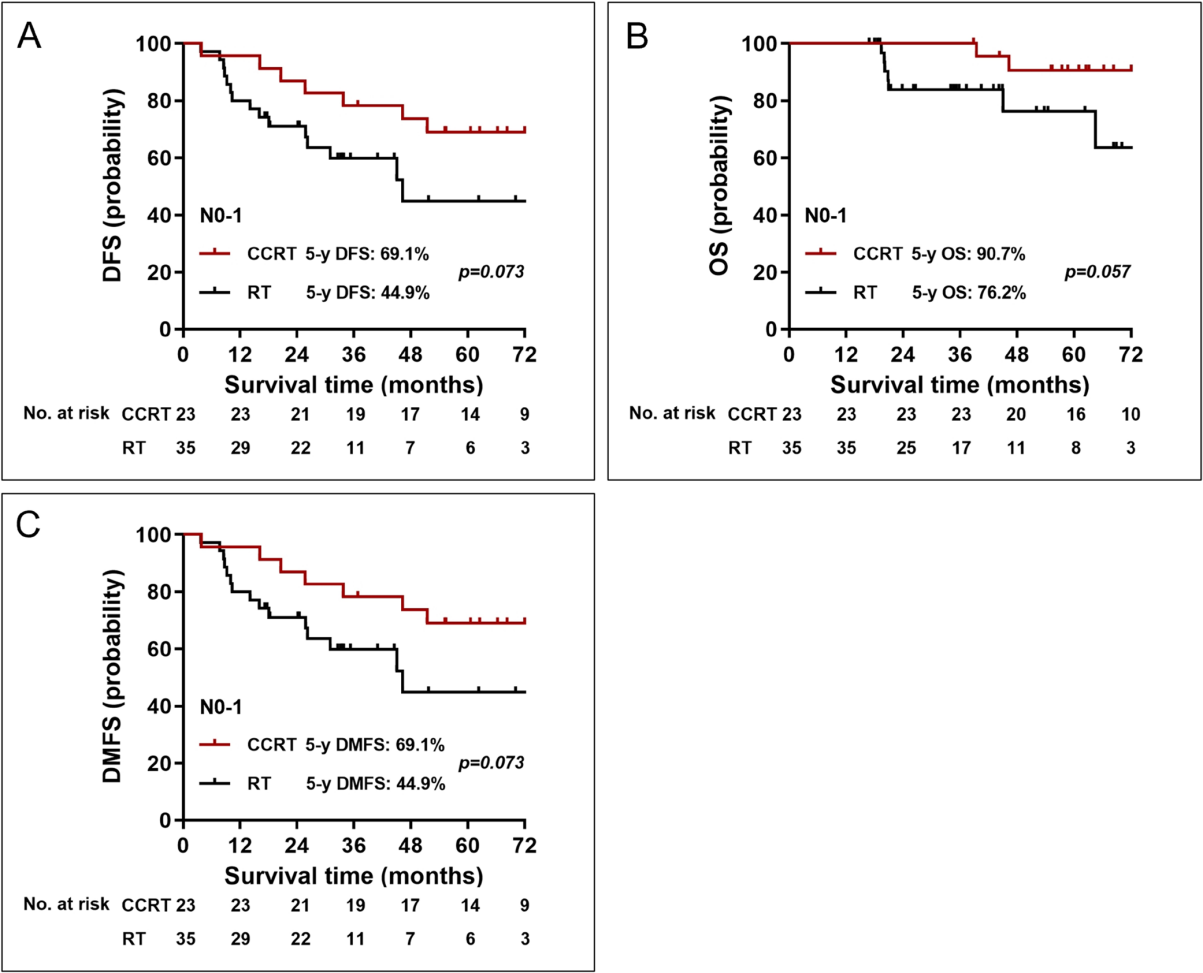
Kaplan–Meier curves between CCRT and RT alone in patients with N0‐1 disease. (A) DFS, (B) OS, (C) DMFS. Abbreviations: CCRT, concurrent chemoradiotherapy; RT, radiotherapy; DFS, disease‐free survival; OS, overall survival; DMFS, distant metastasis‐free survival.

We also observed that patients with N0‐1 disease treated with CCRT showed a numerically higher 5‐year DFS (69.1% vs. 44.9%, *p* = 0.073), 5‐year OS (90.7% vs. 76.2%, *p* = 0.057), and 5‐year DMFS (69.1% vs. 44.9%, *p* = 0.073) compared to those treated with RT alone (Figure 3).

In patients aged ≤ 65 years treated with CCRT, there was a numerically higher 5‐year DFS (50.4% vs. 25.2%, *p* = 0.050), 5‐year DMFS (55.1% vs. 27.2%, *p* = 0.069) compared to those treated with RT alone. In patients with T1‐2 disease treated with CCRT, there was a numerically higher 5‐year LRRFS (75.0% vs. 44.4%, *p* = 0.064). In patients without PNI treated with CCRT, there was a numerically higher 5‐year OS (83.1% vs. 66.7%, *p* = 0.054) (Figure S1). In patients with Ca ex PA, there was a numerically higher 5‐year DFS (53.3% vs. 25.7%, *p* = 0.064), 5‐year LRRFS (68.8% vs. 52.9%, *p* = 0.074), and 5‐year DMFS (57.4% vs. 29.0%, *p* = 0.098) compared to those treated with RT alone (Figure S2).

Based on subgroup analyses indicating that CCRT was associated with improved outcomes in specific cohorts, we performed multivariate analyses to further assess its therapeutic benefit. The results confirmed that for patients without ENE, CCRT was consistently associated with superior survival outcomes, including DFS (*p* = 0.021), OS (*p* = 0.004), LRRFS (*p* = 0.020), and DMFS (*p* = 0.042, Table 3). Furthermore, CCRT was identified as an independent predictor of improved DFS and DMFS in both patients with N0‐1 disease (*p* = 0.027 for both, Table 3) and those with Ca ex PA (*p* = 0.014 and *p* = 0.039, respectively, Table S3).

**TABLE 3:** Multivariable analysis of the association between CCRT and survival outcomes across subgroups (*).

Subgroup (CCRT vs RT)		DFS HR (95% CI), *p*	OS HR (95% CI), *p*	LRRFS HR (95% CI), *p*	DMFS HR (95% CI), *p*
**ENE**	**No (44 vs 50)**	**0.439 (0.218–0.885) *p* = 0.021** *	**0.159 (0.046–0.550) *p* = 0.004** *	**0.318 (0.121–0.836) *p* = 0.020** *	**0.47 0 (0.228–0.972) *p* = 0.042** *
	**Yes (11 vs 11)**	1.541 (0.336–7.068) *p* = 0.578	25.297 (1.775–360.492) *p* = 0.017	4.573 (0.790–26.463) *p* = 0.0.090	1.441 (0.313–6.626) *p* = 0.639
**N stage**	**N0‐1 (23 vs 35)**	**0.280 (0.091–0.864) *p* = 0.027** *	0.195 (0.017–2.211) *p* = 0.187	0.379 (0.069–2.094) *p* = 0.0.266	**0.280 (0.091–0.864) *p* = 0.027** *
	**N2‐3 (32 vs 26)**	0.754 (0.346–1.644) *p* = 0.477	1.261 (0.480–3.313) *p* = 0.638	0.780 (0.336–1.815) *p* = 0.0.565	0.855 (0.382–1.912) *p* = 0.0.702
**Age**	**≤ 65 years (47 vs 44)**	**0.519 (0.267–1.006) *p = 0.052***	0.619 (0.245–1.566) *p = 0.311*	0.555 (0.246–1.252) *p = 0.156*	**0.549 (0.281–1.074) *p = 0.080***
	**> 65 years (8 vs 17)**	0.168 (0.019–1.511) *p* = 0.111	0.325 (0.019–5.460) *p* = 0.435	0.123 (0.06–2.696) *p* = 0.183	0.210 (0.016–2.772) *p* = 0.236
**PNI**	**No (30 vs 30)**	0.752 (0.270–2.093) *p* = 0.585	**0.181 (0.031–1.051) *p* = 0.057**	0.939 (0.228–3.886) *p* = 0.931	0.594 (0.207–1.707) *p* = 0.334
	**Yes (25 vs 31)**	0.691 (0.317–1.505) *p* = 0.352	1.533 (0.552–4.260) *p* = 0.413	0.667 (0.271–1.643) *p* = 0.379	0.771 (0.351–1.693) *p* = 0.517

Abbreviations: Ca ex PA, Carcinoma ex pleomorphic adenoma; CCRT, concurrent chemoradiotherapy; DFS, disease‐free survival; DMFS, distant metastasis‐free survival; ENE, extranodal extension; LRRFS, locoregional recurrence‐free survival; MEC, Mucoepidermoid carcinoma; NOS, not otherwise specified; OS, overall survival; PNI, perineural invasion; ref., reference.

* For all subgroup analyses, the multivariable Cox regression models include the same set of clinically relevant covariates as used in the main analysis, with the exception that the subgroup‐defining variable is excluded from the model when analyzing the respective subgroup.

### 3.5 Safety

In the CCRT cohort, the TP regimen was well tolerated, with no treatment‐related fatalities. Only one patient (1.8%) experienced a grade 4 decrease in white blood cell (WBC) count, and no other grade 4 adverse events (AEs) were observed. The most common grade 3 AEs included oral mucositis (32.7%) and WBC decrease (7.3%). The most frequent grade 1 to 2 AEs were oral mucositis (67.3%), dermatitis (78.2%), WBC decrease (81.8%), anemia (34.5%), dry mouth (16.4%), and vomiting (9.1%). In the CCRT cohort, the most common grade 3 AEs were oral mucositis (24.5%). The most frequent grade 1 to 2 AEs were oral cavity mucositis (70.5%), dermatitis (83.6%), and dry mouth (18.0%). Importantly, all patients successfully completed the radiation treatment in both cohorts.

## 4 Discussion

We present a prospective cohort study evaluating the combination of concurrent TP chemotherapy with IMRT in the management of postoperative high‐risk non‐ACC SGC patients, compared to a retrospective cohort. Although no significant survival advantage was demonstrated in the overall patient population, subgroup analysis revealed an unexpected benefit among patients without ENE. In this subgroup, the integration of concurrent chemotherapy with radiotherapy was associated with improved 5‐year outcomes across multiple endpoints, including OS, DFS, LRRFS, and DMFS rates. Additionally, our study highlighted a significant association between CCRT and improved 5‐year DFS and DMFS in patients with N0‐1 disease and Ca ex PA, compared to radiotherapy alone.

At present, there is a paucity of active randomized trials assessing postoperative CCRT in the treatment of high‐risk SGCs. In two ongoing phase III trials, RTOG 1008 (NCT01220583) and GORTEC SANTAL (NCT02998385), postoperative high‐risk patients are being randomly assigned to a two‐arm study receiving either radiotherapy alone or a combination of radiotherapy and cisplatin. When comparing the main inclusion criteria, the target patient population of the RTOG 1008 and GORTEC SANTAL trial closely resembles that of our study, with the exception that our study exclusively enrolled patients with stage III‐IV disease, which represents a higher‐risk cohort. The chemotherapy protocol utilized in our study diverges from that of the RTOG 1008 and GORTEC SANTAL trials. Instead of employing a single‐agent cisplatin regimen, our study administers a combination of docetaxel (70 mg/m2) and nedaplatin (70 mg/m2) every 3 weeks in conjunction with IMRT. Several factors informed our decision to select this regimen: (1) Combinations of platinum and taxane‐based chemotherapy are widely accepted for managing recurrent/metastatic non‐ACC SGCs, providing a solid foundation for our treatment approach [4, 10, 22, 23, 24]. (2) Docetaxel and nedaplatin exert their effects through distinct mechanisms [25, 26]. The synergy between these agents, when combined with IMRT (TP plus IMRT), is anticipated to enhance local disease control. (3) The triweekly administration of a multi‐agent regimen with high peak doses is designed to more effectively target micrometastases and thereby reduce the likelihood of distant metastases [27]. This approach is pivotal for minimizing disease recurrence and improving long‐term outcomes. (4) The typical target volume for SGCs encompasses the unilateral head and neck region [28], making it feasible to implement a doublet chemotherapy regimen without compromising efficacy.

Although no overall survival benefit was observed in all patients, we found that the subgroup of patients without ENE or with N0‐1 disease may benefit from CCRT. In contrast, patients with ENE or N2‐3 disease did not show any survival benefit from CCRT when compared to RT alone. To compare long‐term outcomes, Heish et al. [18] recently conducted a multicenter, retrospective study in postoperative SGC patients treated with either radiotherapy alone or concurrent chemoradiotherapy (CCRT). The findings suggested that CCRT may enhance long‐term OS and PFS rates in patients with lymph nodal metastatic SGCs compared to RT alone. Additionally, the inclusion of concurrent chemotherapy was linked to better long‐term local‐regional control rates in patients who underwent R2 resection. However, our study revealed that patients with R2 resection did not experience improvements in LRRFS or other survival benefits from CCRT compared with RT alone. A potential explanation for this discrepancy could lie in the differences in radiation therapy techniques used. In our study, IMRT, which provides superior dose distribution and precision, was universally adopted. This contrasts with the study by Heish et al. [18], where some patients received three‐dimensional conformal radiation therapy (3D‐CRT), which lacks the same level of precision and dosimetric advantages as IMRT. The reduced efficacy of CCRT in our cohort may, therefore, be related to the potential limitations in the radiation technique, rather than the modality itself.

Multivariate analysis demonstrated that patients with ENE or N2‐3 disease are at a very high risk of worse DFS and OS. Since ENE is well established as a high‐risk factor and an indication for CCRT in postoperative head and neck squamous carcinoma [29, 30, 31], its prognostic role in SGCs remains disputed and has not been well defined [32, 33, 34, 35]. Our study showed that patients with ENE had a very poor prognosis, with a 5‐year DFS rate of only 8.6%, which is consistent with the findings of Lee H et al. [33], and Hsieh CE et al. [32]. Our study reveals that nodal status also acts as an important prognostic factor, and patients with N2‐3 disease had a 5‐year DFS of 27.9%, which is consistent with the previous findings [34, 35]. However, our findings indicated that these very high‐risk subgroups did not experience additional benefits from CCRT compared to RT alone. This lack of benefit may suggest the limited efficacy of CCRT in improving outcomes for patients with extensive disease burden or very high‐risk pathological features. It underscores the need for a paradigm shift in treatment strategies for these patients. Further research is required to explore alternative therapeutic approaches, such as adjuvant androgen deprivation therapy [36], adjuvant anti‐Her2 therapy [37] (NCT05087706 and NCT04620187) to improve survival outcomes in these high‐risk populations.

The study's primary limitation is its relatively small sample size, a common challenge in SGC research due to the rarity of these cases. This constraint compromises the robustness of subgroup analyses, which are needed to elucidate the efficacy of CCRT in specific patient cohorts—such as those with ENE, R2 resection, LVI, or particular histologic subtypes (e.g., MEC, adenocarcinoma, SDC). Another limitation is that the control cohort was retrospectively collected and with shorter follow‐up time, which may introduce potential biases. The use of the Cox model strengthens the analysis by mitigating the impact of confounding factors and helping to clarify the role that differences in follow‐up time may play in influencing the study's results. Thirdly, the lacking of universally accepted grading system for some certain entities potentially affecting the interpretation of treatment outcomes. Finally, analyses of secondary endpoints and subgroups are considered exploratory and should be interpreted with caution. Despite these limitations, we believe it is appropriate to present these data, given the paucity of prospective evidence available regarding this treatment approach.

## 5 Conclusions

For postoperative high‐risk non‐adenoid cystic carcinoma (non‐ACC) SGCs, concurrent IMRT and TP chemotherapy were associated with improved long‐term survival outcomes in patients without ENE or with N0‐1 disease. However, for patients with ENE or N2‐3 disease, who are at very high risk for recurrence, the benefits of CCRT were less pronounced. This suggests that these patients may require a more aggressive or alternative treatment strategy to better manage their prognosis. Large‐scale randomized controlled trials with extended follow‐up are required to validate these results and optimize treatment strategies for high‐risk SGC patients.

## Author Contributions

**Shengjin Dou:** conceptualization (equal), data curation (equal), formal analysis (equal), investigation (equal), methodology (equal), project administration (equal), visualization (equal), writing – original draft (lead), writing – review and editing (equal). **Xin Wang:** data curation (equal), investigation (equal), resources (equal), writing – review and editing (equal). **Lin Zhang:** resources (equal), writing – review and editing (equal). **Wen Jiang:** resources (equal), writing – review and editing (equal). **Lulu Ye:** resources (equal), writing – review and editing (equal). **Yu Wang:** resources (equal), writing – review and editing (equal). **Rongrong Li:** methodology (equal), resources (equal), supervision (equal), validation (equal), writing – review and editing (equal). **Guopei Zhu:** conceptualization (equal), funding acquisition (equal), resources (equal), supervision (equal), validation (equal), writing – review and editing (equal).

## Funding

This study was supported by Shanghai Municipal Health Commission (201640158).

## Ethics Statement

The study was performed in accordance with the Declaration of Helsinki. This study was approved by the Institutional Review Board of Shanghai Ninth People's Hospital.

## Consent

Informed consent was obtained from participants in the prospective cohort, while it was waived for the retrospective cohort.

## Conflicts of Interest

The authors declare no conflicts of interest.

## Supporting information


**Data S1:** Supplementary Information.

## Data Availability

The data that support the findings of this study are available from the corresponding author upon reasonable request.
