# Supplementary material for: Long‐Term Survival Outcomes of Concurrent Chemoradiotherapy for Postoperative High‐Risk Salivary Gland Carcinomas
Source: Cancer Med. 2026 Mar 25;15(3):e71722. doi: 10.1002/cam4.71722 (PMC13140899; doi:10.1002/cam4.71722)
Supplement: Supplementary file 1 — Data S1: Supplementary Information. [file CAM4-15-e71722-s001.docx]

**Supplementary figure**


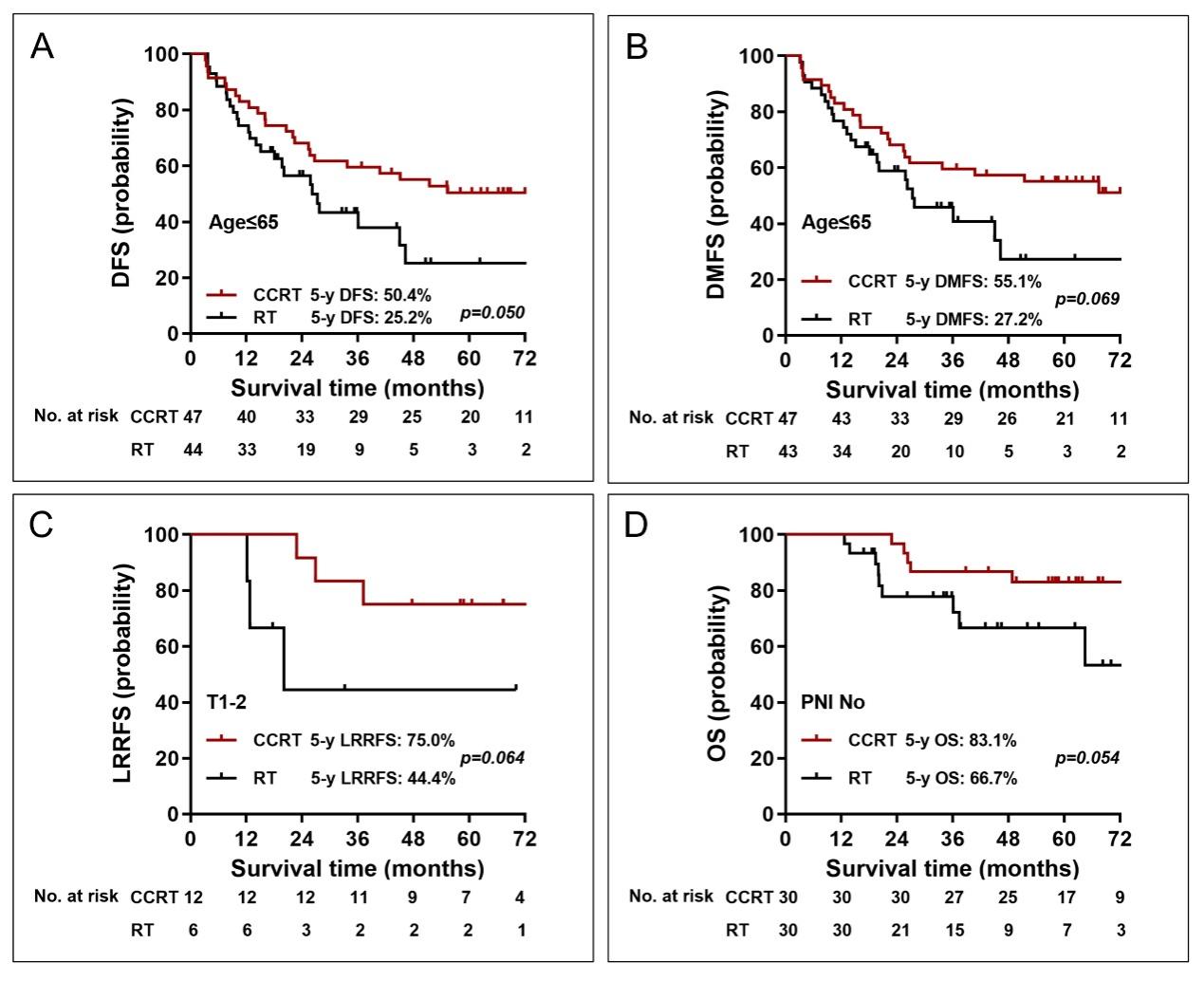


**Supplementary eFigure 1.** Kaplan–Meier curves between CCRT and RT alone in subgroups. (A) DFS, and (B), DMFS in patients with age≤65 years, (C) LRRFS in patients with T1-2 disease and (D) OS in patients without PNI. Abbreviations：CCRT, concurrent chemoradiotherapy; RT, radiotherapy; DFS, disease-free survival; OS, overall survival; LRRFS, locoregional recurrence-free survival; DMFS, distant metastasis-free survival; PNI, perineural invasion.


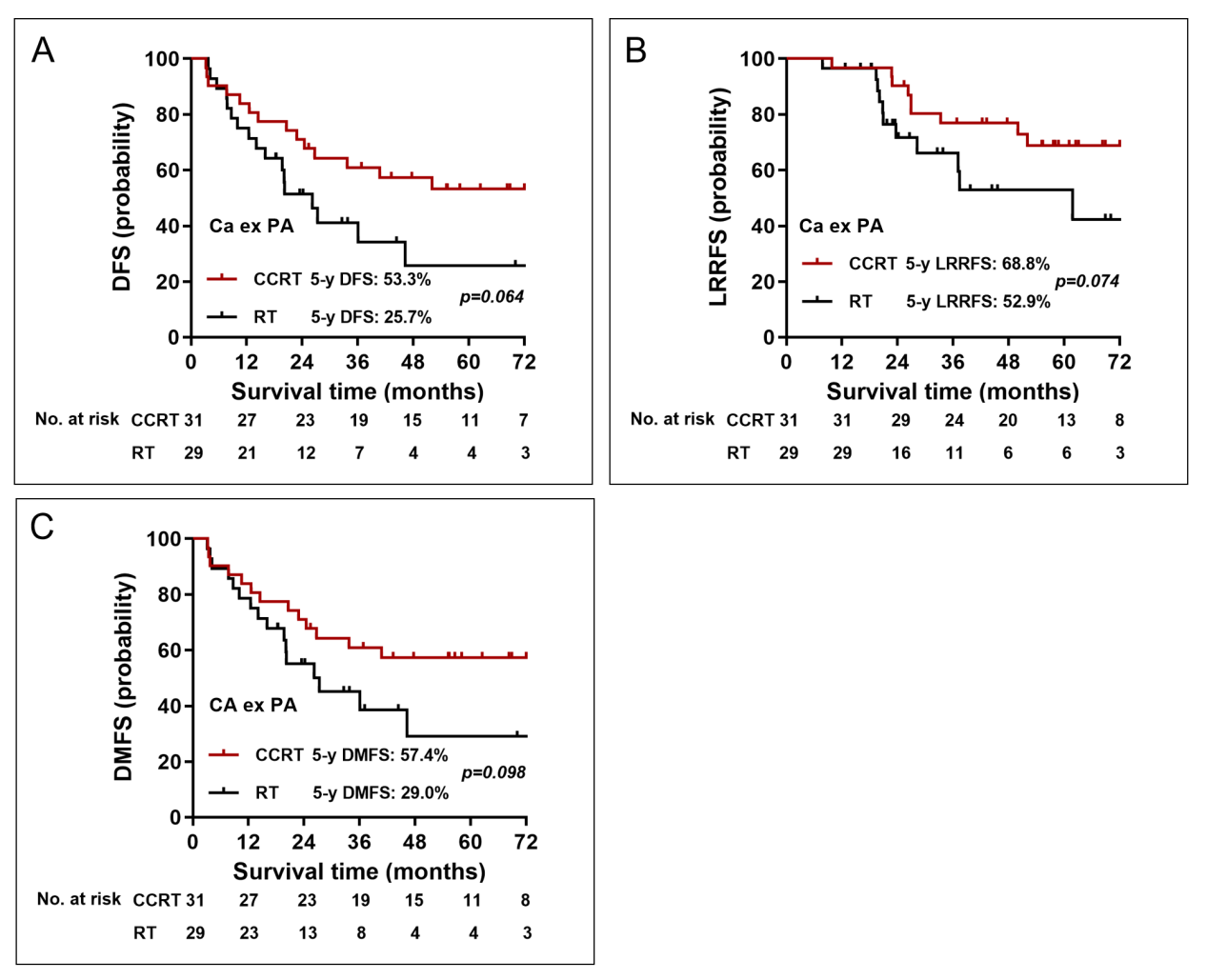


**Supplementary eFigure 2.** Kaplan–Meier curves between CCRT and RT alone in Ca ex PA subgroup. (A) DFS, (B) LRRFS and (C) DMFS in patients with Ca ex PA . Abbreviations：CCRT, concurrent chemoradiotherapy; RT, radiotherapy; DFS, disease-free survival; LRRFS, locoregional recurrence-free survival; DMFS, distant metastasis-free survival; Ca ex PA, Carcinoma ex pleomorphic adenoma.

**Supplementary eTable 1.** Univariable analysis of all patients.

| **All patients (n=116)** | | **5-year DFS** | **5-year OS** | **5-year LRRFS** | **5-year DMFS** |
| --- | --- | --- | --- | --- | --- |
| **CCRT** | **Yes vs No (ref)** | 47.6% vs 37.5% *(p=0.252)* | 70.0% vs 67.4% *(p=0.284)* | 62.1% vs 59.3% *(p=0.252)* | 53.8% vs 39.9% *(p=0.332)* |
| **Sex** | **Female vs Male (ref)** | 48.8% vs 40.2% *(p=0.450)* | 72.1% vs 67.0% *(p=0.708)* | 67.9% vs 57.1% *(p=0.488)* | 53.6% vs 46.2% *(p=0.499)* |
| **Age (years)** | **＞65 vs ≤65 (ref)** | 46.0% vs 40.7% *(p=0.258)* | 73.6% vs 67.3.% *(p=0.885）* | 60.6% vs 59.5% *(p=0.969）* | 60.1% vs 45.1% *(p=0.127)* |
| **Site** | **Minor vs Major (ref)** | 49.5% vs 40.4% *(p=0.330)* | **91,1% vs 61.6% *(p=0.012*)*** | **76.3% vs 54.8% *(p=0.065)*** | 45.2% vs 59.4% *(p=0.154)* |
| **T stage** | **T3-4 vs T1-2 (ref)** | 39.4% vs 54.2% *(p=0.485)* | 68.8% vs 67.7% *(p=0.795)* | 58.5% vs 64.6% *(p=0.722）* | 45.9% vs 58.3% *(p=0.449)* |
| **N stage** | **N2-3 vs N0-1 (ref)** | **27.9% vs 56.4% *(p=0.002*)*** | **54.1% vs 82.6% *(p=0.002*)*** | **41.2% vs 79.5% *(p=0.000*)*** | **40.0% vs 56.4% *(p=0.014*)*** |
| **Grade** | **III vs II (ref)** | 40.7% vs 50.8% *(p=0.220)* | 67.6% vs 72.1% *(p=0.519)* | 58.9% vs 65.0% *(p=0.360）* | 46.3% vs 57.1% *(p=0.206)* |
| **Surgery Status** | **R2 vs R0R1 (ref)** | **24.6% vs 44.7% *(p=0.094)*** | 61.5% vs 69.2% *(p=0.601)* | 52.9% vs 60.7% *(p=0.464）* | **24.6% vs 51.4% *(p=0.058)*** |
| **ENE** | **Yes vs No (ref)** | **8.6% vs49.6% *(p=0.000*)*** | **30.5% vs 76.5% *(p=0.000*)*** | **25.6% vs 67.3% *(p=0.000*)*** | **8.6% vs 56.1% *(p=0.000*)*** |
| **LVI** | **Yes vs No (ref)** | **14.1% vs 46.8% *(p=0.028*)*** | 38.4% vs 73.0% *(p=0.122)* | **22.6% vs 65.7% *(p=0.004*)*** | **15.6% vs 52.8% *(p=0.051)*** |
| **PNI** | **Yes vs No (ref)** | **33.6% vs 50.6% *(p=0.065)*** | 58.7% vs 75.6% *(p=0.165）* | 52.7% vs 66.2% *(p=0.144）* | **35.9% vs 58.8% *(p=0.087)*** |

Abbreviations: CCRT, concurrent chemoradiotherapy; RT, radiotherapy; LVI, lymph-vascular invasion; PNI, perineural invasion; ENE, extranodal extension; DFS, disease-free survival; OS, overall survival; LRRFS, locoregional recurrence-free survival; DMFS, distant metastasis-free survival.

Supplementary eTable 2. Univariable analysis of the association between CCRT and survival outcomes across subgroups

| **Subgroup**  **(****CCRT vs RT)** | | **5-year DFS** | **5-year OS** | **5-year LRRFS** | **5-year DMFS** |
| --- | --- | --- | --- | --- | --- |
| **Sex** | **Male (42 vs 46)** | 46.0% vs 32.0% *(p=0.305)* | 65.2% vs 70.0% *(p=0.760)* | 57.1% vs 60.0% *(p=0.478)* | 51.7% vs 34.9% *(p=0.490)* |
|  | **Female (13 vs 15)** | 53.8% vs 48.6% *(p=0.443)* | 84.6% vs 60.0% *(p=0.113)* | 76.9% vs 58.3% *(p=0.342)* | 61.5% vs 48.6% *(p=0.307)* |
| **Age** | **≤65 years（47 vs 44）** | **50.4% vs 25.2% *(p=0.050)*** | 71.8% vs 60.4% *(p=0.136)* | 65.1% vs 50.8% *(p=0.099)* | **55.1% vs 27.2% *(p=0.069)*** |
|  | **＞65 years (8 vs 17)** | 23.4% vs 67.9% *(p=0.327)* | 60.0% vs 85.7% *(p=0.501)* | 40.0% vs 80.7% *(p=0.420)* | 46.9% vs 72.5% *(p=0.381)* |
| **Site** | **Major (41 vs 50)** | 49.4% vs 31.2% *(p=0.080)* | 61.7% vs 63.7% *(p=0.432)* | 55.8% vs 58.1% *(p=0.294)* | 55.4% vs 32.2% *(p=0.092)* |
|  | **Minor (14 vs 11)** | 42.9% vs 68.2% *(p=0.364)* | 92.9% vs 87.5% *(p=0.741)* | 78.6% vs 66.7% *(p=0.792)* | 50.0% vs 77.8% *(p=0.179)* |
| **T stage** | **T1-2 (12 vs 6)** | 58.3% vs 50.0% *(p=0.393)* | 75.0% vs 37.5% *(p=0.209)* | **75.0% vs 44.4% (p=0.064)** | 58.3% vs 66.7% *(p=0.829)* |
|  | **T3-4 (43 vs 55)** | 44.4% vs 35.7% *(p=0.311)* | 68.7% vs 70.4% *(p=0.488)* | 58.3% vs 61.0% *(p=0.565)* | 52.5% vs 37.4% *(p=0.311)* |
| **N stage** | **N0-1 (23 vs 35)** | **69.1% vs 44.9% *(p=0.073)*** | **90.7% vs 76.2% *(p=0.057)*** | 86.5% vs 72.6% *(p=0.148)* | **69.1% vs 44.9% *(p=0.073)*** |
|  | **N2-3 (32 vs 26)** | 31.8% vs 26.0% *(p=0.387)* | 55.0% vs 54.4% *(p=0.531)* | 44.0% vs 40.3% *(p=0.203)* | 43.3% vs 33.1% *(p=0.662)* |
| **Grade** | **II (10 vs 11)** | 50.0% vs 51.1% *(p=0.910)* | 80.0% vs 63.6% *(p=0.476)* | 68.6% vs 60.6% *(p=0.680)* | 60.0% vs 51.1% *(p=0.906)* |
|  | **III (45 vs 50)** | 47.0% vs 34.3% *(p=0.182)* | 67.9% vs 70.6% *(p=0.407)* | 60.6% vs 61.0% *(p=0.310)* | 52.3% vs 37.4% *(p=0.295)* |
| **Surgery status** | **R0R1 (50 vs 53)** | 50.6% vs 38.6% *(p=0.158)* | 71.0% vs 69.8% *(p=0.210)* | 64.2% vs 60.0% *(p=0.100)* | 57.5% vs 41.6% *(p=0.234)* |
|  | **R2 (5 vs 8)** | 20.0% vs 33.3% *(p=0.468)* | 60.0% vs 55.6% *(p=0.695)* | 40.0% vs 55.6% *(p=0.216)* | 20.0% vs 33.3% *(p=0.468)* |
| **ENE** | **No (44 vs 50)** | **57.3% vs 41.6% (*p=0.062)*** | **83.4% vs 69.0% *(p=0.032*)*** | **73.4% vs 62.3% *(p=0.071)*** | **65.2% vs 44.5% *(p=0.089)*** |
|  | **Yes (11 vs 11)** | 0.91% vs 0.0% *(p=0.599)* | 18.2% vs 63.6% *(p=0.265)* | 18.2% vs 43.3% *(p=0.527)* | 0.91% vs 0.0% *(p=0.680)* |
| **LVI** | **No (49 vs 51)** | 51.7% vs 41.5% *(p=0.270)* | 72.9% vs 74.9% *(p=0.459)* | 65.9% vs 68.0% *(p=0.463)* | 58.7% vs 43.2% *(p=0.293)* |
|  | **Yes (6 vs 10)** | 16.7% vs 25.0% *(p=0.756)* | 50.0% vs 38.9% *(p=0.265)* | 33.3% vs 28.1% *(p=0.276)* | 16.7% vs 30.0% *(p=0.949)* |
| **PNI** | **No (30 vs 30)** | 54.6% vs 51.3% *(p=0.419)* | **83.1% vs 66.7% *(p=0.054)*** | 68.6% vs 65.2% *(p=0.349)* | 66.1% vs 51.3% *(p=0.302)* |
|  | **Yes (25 vs 31)** | 38.6% vs 24.9% *(p=0.491)* | 54.1% vs 63.8% *(p=0.713)* | 54.1% vs 51.1% *(p=0.543)* | 38.6% vs 28.5% *(p=0.804)* |
| **Pathology*** | **Ca ex PA(31 vs 29)** | **53.3% vs 25.7% *(p=0.064)*** | 73.2% vs 70.0% *(p=0.332)* | **68.8% vs 52.9% *(p=0.074)*** | **57.4% vs 29.0% *(p=0.098)*** |
|  | **MEC(11 vs 13)** | 72.7% vs 51.9% *(p=0.220)* | 90.9% vs 66.6% *(p=0.160)* | 81.8% vs 65.3% *(p=0.343)* | 81.8% vs 51.9% *(p=0.135)* |
|  | **Adenocarcinoma, NOS(8 vs 9)** | 0.0% vs 0.0% *(p=0.439)* | 37.5% vs 37.5% *(p=0.842)* | 25.0% vs 44.4% *(p=0.458)* | 0.0% vs 0.0% *(p=0.439)* |

Abbreviations: CCRT, concurrent chemoradiotherapy; RT, radiotherapy; LVI, lymph-vascular invasion; PNI, perineural invasion; ENE, extranodal extension; DFS, disease-free survival; OS, overall survival; LRRFS, locoregional recurrence-free survival; DMFS, distant metastasis-free survival; Ca ex PA, Carcinoma ex pleomorphic adenoma; MEC, Mucoepidermoid carcinoma; NOS, not otherwise specified.

*Subgroups with fewer than 5 patients were not shown.

Supplementary eTable 3. Multivariable analysis of the association between CCRT and survival outcomes in pathology subgroups

| **Subgroup**  **(CCRT vs RT)** | | **DFS** | **OS** | **LRRFS** | **DMFS** |
| --- | --- | --- | --- | --- | --- |
|  |  | **HR (95%CI), *p*** | **HR (95%CI), *p*** | **HR (95%CI), *p*** | **HR (95%CI), *p*** |
| **Pathology*** | **Ca ex PA(31 vs 29)** | **0.356(0.156-0.815)**  ***p=0.014**** | 0.893(0.265-3.008)  *p=0.855* | 0.470(0.164-1.346)  *p=0.159* | **0.421(0.185-0.957)**  ***p=0.039**** |
|  | **MEC(11 vs 13)** | 0.097(0.006-1.628)  *p=0.105* | 0.000(0.000-3.822E+139)  *p=0.910* | 0.000(0.000-1.283E+115)  *p=0.932* | 0.102(0.007-1.521)  *p=0.098* |
|  | **Adenocarcinoma, NOS(8 vs 9)** | 1.908(0.113-32.267)  *p=0.654* | 47.896.880(0.000-5.593E+179)  *p=0.958* | 12.267(0.009-16092.503)  *p=0.494* | 1.908(0.113-32.267)  *p=0.654* |

*Subgroups with fewer than 5 patients were not shown.
